# A Decision against Healers

**Published:** 1899-10

**Authors:** 


### A DECISION AGAINST HEALERS.

THE christian science, Zion curers, faith healers of Chicago have run up against a learned judge at last. The following are the circumstances of the case:

Mrs. Flanders, into the circumstances of whose death the investigation was made, died at St. Luke's Hospital of blood poisoning, which the physicians which were called to attend the woman just before she died assert was the result of medical and criminal neglect. Mrs. Flanders was a believer in "divine healing," and a member of the Divine Healing Mission in Michigan avenue, near Sixteenth street, of which institution Dr. Dowie calls himself "general overseer."

Nearly two weeks ago, on being taken critically ill, Mrs. Flanders requesting that Mrs. Bratz be summoned to her bedside to administer "divine healing." According to Mrs. Bratz nothing was done to relieve her patient's sufferings by physical means. Mrs.

Bratz, however, in accordance with the belief, said prayers, and when it was found that Mrs. Flanders was getting worse Dr. D. C. Holmes was called from the Zion Home to assist in the praying.

A few days later the patient became delirious. After a week of "divine healing" Mrs. Flanders was still worse and Dr. H. D. Peterson was called. It was he who first called the attention of the State Board of Health to the matter. He asserts that Mrs. Flanders was delerious and was suffering from blood poisoning when he arrived at the Flanders home. Removal to St. Luke's Hospital was ordered as the only chance of saving Mrs. Flander's life. At the hospital she was found to be beyond medical aid and dying.

Dr. Peterson testified that the blood poisoning of Mrs. Flanders was the result directly of medical negligence.

The defense of the Dowie officials, as given out by Dr. Speicher was that Mrs. Flanders died at the hospital and when not in the care of Mrs Bratz.

The case came to trial August 16th, when Mrs. Bratz, was fined $100 and costs in Justice Everett's court for practising medicine without a license. She was the nominal defendant, but the center of interest to the spectators and the one whom the decision affects even to a greater degree was Dr. John Alexander Dowie. If Justice Everett's opinion is sustained in the higher court the leader of the Zion movement, lawyers say, no longer can cure by "the laying on of hands." An appeal was taken to the Circuit Court. John Stearns signed the $250 bonds.

The defense introduced no witnesses, but asked that the case be dismissed on four grounds, including that Mrs. Bratz did not act as a physician, that the law forbade any interference with those who believed in the efficacy of faith cures and that neither the state nor the nation had any right to interfere with the practice of any religion.

These grounds, on which the leader and members of the christian catholic church based their defense, were admittedly points which a justice court cannot decide and which must be carried higher.

In his decision Justice Everett said :

There is no question as to the right of every man to have and to hold his own religious belief and no question as to the right of a man to call upon whomsoever he sees fit to treat him during sickness, no matter what school of medicine or school of treatment the person called upon belongs.

There is no question but that a man can call in a person to treat him who resorts to spiritual or mental means to cause him to recover his health. But the law does say that, where a person resorts to the use of medicine, because the use of drugs is dangerous, the person shall have a license to practise. There is no question but that the members of this faith can treat within the meaning of the faith as long as the faith does not overlap the law.

If Mrs. Bratz treated Mrs. Flanders by mental or spiritual means without the use of drugs or material remedies she is innocent. The whole case simmers down to this point. Even if a person does not use drugs or material remedies and does not limit himself or herself to spiritual or mental means they are, in my opinion, clearly liable. When the defendent resorted to physical means of any kind by putting her hands on the decedent then she is liable under the law.

The line must be drawn sharply and right at the spiritual end and stop there. In this case the defendant fell back upon her experience and not upon her faith and resorted to physical means. I have nothing against the followers of the faith of Zion. I have noticed in the whole course of history that when the civil courts endeavor to disparage the tenets of any religious faith the courts have been worsted.

I believe Pontius Pilate was the first to make such an attempt. I do not desire to emulate him, although in this matter I do not intimate that I have the same object before me. The defendant is fined $100 and costs.

We gather the foreging from the Chicago *Tribune* of recent date. The *Tribune* also published an able editorial concerning the death of Mrs. Flanders, which we would be glad to reproduce did space permit.

---

### A MEDICAL LAW FOR MICHIGAN.

By the (*Medical Age*, *Detroit*,) terms of the Chandler bill, which by the signature of the governor becomes a law, the State of Michigan will reap the advantage of a measure which is as satisfactory as that which is operative in any other state of the Union. The terms of this enactment include the following provisions:

1. The appointment of a medical board of registration composed of five regulars, two homeopaths, two eclectics, and one physiomedicist. The members of this board, which meets twice each year at Lansing, form the executive force of the law.

2. The reregistration of all physicians in the state who furnish proof to the board that they are legally registered under the act of 1883. This is expected to shut out the holders of bogus and fraudulent diplomas, and was bitterly fought by some so-called medical "colleges" for that reason.

3. The recognition of foreign certificates of registration or licenses issued where the requirements for registration are at least equal to those required here; but the county or state so recognised must extend a like privilege to holders of certificates from the Michigan board.

4. The recognition of diplomas from such legally incorporated and reputable colleges of medicine in the United States as shall be approved for in the foregoing.

6. The reporting monthly to the board by the several county clerks in the state of the registration made, removals and death.

7. The enforcement of the act by the board direct, not by local authority, as under the old act.

